# Efficient PE system PE7-scFv-MLH1dn by optimizing the configuration of La and MLH1dn

**DOI:** 10.1016/j.synbio.2026.04.035

**Published:** 2026-05-25

**Authors:** Xuesong Li, Chaojun Qing, Bo Li, Dongdong Zhao, Peihua Li, Wenzhu Tang, Changhao Bi, Xueli Zhang

**Affiliations:** a School of Biological Engineering, Dalian Polytechnic University, Dalian, 116034, China; b Chinese Academy of Sciences, Tianjin Institute of Industrial Biotechnology, Tianjin, 300308, China; c National Center of Technology Innovation for Synthetic Biology, Tianjin, 300308, China; d College of Biotechnology, Tianjin University of Science and Technology, Tianjin, 300222, China; e Department of Medical Genetics, West China Second University Hospital, Sichuan University, Chengdu, Sichuan, 610041, China

**Keywords:** Gene editing, Prime editing, Recruitment, Auxiliary factor, MLH1dn

## Abstract

Prime editing is a versatile gene editing technology that has undergone significant enhancements since its inception. Currently, a recently reported prime editing system, PE7 combined with MLH1dn, incorporates two auxiliary components designed to enhance editing efficiency: La fused to the reverse transcriptase (RT) and non-recruited MLH1dn. We hypothesized that the expression and recruitment strategies for these proteins could be further optimized. In this study, we first employed the SunTag system to recruit La and MLH1dn, either individually or simultaneously, and found that recruitment, particularly co-recruitment, significantly enhanced prime editing efficiency. Subsequently, we evaluated various expression and recruitment configurations and determined that a PE system utilizing 2∗GCN4-anchored scFv-fused MLH1dn recruitment, while retaining La fused to RT, achieved the highest editing efficiency. Testing across multiple genomic loci demonstrated that this auxiliary factor–based strategy further improved editing efficiency compared with PE7 alone. In summary, by systematically exploring the recruitment modalities of auxiliary factors, we have developed a more efficient prime editing system, providing researchers with a state-of-the-art tool for high-efficiency genome modification.

## 1 Introduction

Clustered regularly interspaced short palindromic repeats (CRISPR) and CRISPR-associated (Cas) proteins constitute a widespread system in microbial genomes and play a central role in adaptive immunity against viral infection in bacteria [[1], [2], [3]]. Repurposing the RNA-guided DNA-cleavage activity of this system led to the development of CRISPR–Cas9 genome editing technology [[4], [5], [6], [7]]. Since the advent of CRISPR–Cas9, extensive efforts to reengineer the system have driven the emergence of base editors (BEs) and prime editors (PEs) [[8], [9], [10], [11], [12]]. Among these genome editing tools, prime editors are the most recently developed and offer highly versatile editing capabilities, enabling a broad spectrum of base conversions, including four types of base transitions and eight types of base transversions, as well as small insertions and deletions within the genome [12]. Prime editing has therefore attracted considerable interest for therapeutic applications (for example, cell- and gene-therapy) and for disease modeling [13,14].

PE2 is the originally optimized prime editor, consisting of an engineered protein that fuses Cas9 H840A nickase with a reverse transcriptase, together with a pegRNA that specifies both the DNA target and the intended edit [12,15]. The working model of prime editing is as follows: The prime editing protein binds the pegRNA to form a prime editing complex, which is guided by the pegRNA spacer to locate the complementary DNA target. Upon target recognition, the complex nicks one DNA strand and releases a 3′ DNA end. This 3′ end hybridizes to the pegRNA's 3′ extension (which contains the primer-binding site and the reverse-transcription template) and primes reverse transcription using the pegRNA as the template, thereby synthesizing an extended 3′ DNA strand that encodes the intended edit. The newly synthesized 3′ DNA strand bearing the edit is ultimately preserved and incorporated into the genome [16,17].

However, prime editing efficiency varies widely across different edit types, genomic loci, and cell types, and is often limited, which restricts its broader applicability [18,19]. Consequently, substantial efforts have been devoted to developing enhanced prime editing systems. Recent studies indicate that directed recruitment of auxiliary factors via engineered scaffolds is an effective and broadly applicable strategy to boost editing performance [12,20]. These approaches generally fall into three categories. First, modulation of DNA repair pathways, such as transient inhibition of mismatch repair or biasing repair toward precise outcomes, can stabilize reverse-transcribed intermediates and increase their retention [17,18,21]. Second, chromatin and transcriptional regulation–assisted strategies enhance local DNA accessibility without directly perturbing core repair pathways [[22], [23], [24]]. Third, multivalent scaffold-based recruitment systems, including RNA–protein or peptide-based platforms, locally enrich effector factors at target sites, thereby increasing their effective concentration and residence time [3,24,25]. Together, these findings highlight auxiliary factor recruitment as a mechanistically grounded route for improving precise genome editing. Two such cellular factors have been identified that significantly enhance prime editing efficiency, leading to the development of the PE4 and PE7 prime editing systems [26,27]. Chen and colleagues discovered that mismatch repair (MMR) activity strongly suppresses the efficiency of replacement-type prime edits, providing the rationale for developing the PE4 system [16]. They investigated the co-delivery of prime editors with MMR inhibitors and found that a dominant-negative variant of the MMR protein MLH1 (MLH1Δ*754–756*, termed MLH1dn) could more effectively enhance prime editing efficiency. Their experiments demonstrated that MLH1dn inhibits MMR either by forming a catalytically impaired MutLα complex with PMS2 or by saturating MSH2 binding, thereby increasing editing efficiency in a dose-dependent manner. The prime editing system co-delivering PE2 with MLH1dn was thus designated PE4.

Jun Yan and colleagues identified La, a small RNA-binding protein, as a potent enhancer of prime editing efficiency [27]. They showed that fusing the N-terminal La domain (La1–194) of full-length La to the C-terminus of the PEmax prime editor created a construct with significantly improved editing efficiency, termed PE7. Their experiments indicated that La functionally interacts with the 3′ end of polyuridylated pegRNAs and promotes the stability and integrity of Pol III-transcribed pegRNAs. Consequently, the PE7 system used in conjunction with pegRNAs, which was reported to outperform PEmax under a wide range of tested conditions, represents a strong foundation for further optimization.

The discovery and application of the cellular factors MLH1dn and La(1–194) have advanced the development of prime editing systems and provided conceptual frameworks that inform the mechanisms and rational design of prime editing. In the studies described above, the two factors enhance prime editing efficiency via distinct mechanisms and are employed differently: La(1–194) is fused directly to the PEmax prime editor, whereas MLH1dn is expressed freely.

In this study, we sought strategies to achieve higher prime editing efficiency by leveraging both cellular factors. We found that recruiting the cellular factor MLH1dn using a PE7 prime editor fused with a SunTag-based recruitment system could further enhance editing efficiency. Based on this observation, we modified the conventional approach of freely overexpressing MLH1dn and instead employed a recruitment strategy to achieve higher prime editing efficiency.

## 2 Result

### 2.1 Recruitment of MLH1dn, La(1–194), or La enhances PEmax editing efficiency

**Fig. 1:**
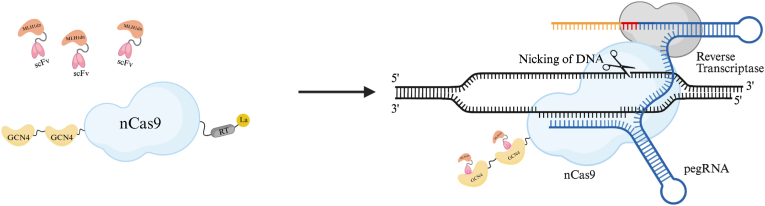
Schematic of the mechanism of PE7-scFv-MLH1dn with GCN4–scFv based MLH1dn recruitment system for enhanced prime editing.

We hypothesized that recruiting higher local concentrations of the cellular factors MLH1dn, La(1–194), or La near the editing site could enhance prime editing efficiency compared with non-recruited expression of these factors. To achieve this, we adopted the strategy previously reported by Ronghao Chen and colleagues, who used the SunTag system in the PE2 platform to recruit the P65 protein [23]. Using the same approach, we successfully constructed the following plasmids: 2∗GCN4-PEmax, 2∗GCN4-PEmax-P2A-MLH1dn, and 2∗GCN4-PE7 prime editors, as well as scFv-MLH1dn, scFv-La(1–194), and scFv-La. In this system, the GCN4 peptide is specifically recognized by a single chain variable fragment (scFv), enabling the recruitment of multiple scFv-MLH1dn or scFv-La(1–194) fusion proteins in close proximity to the editing site (Fig. 1).

**Fig. 2:**
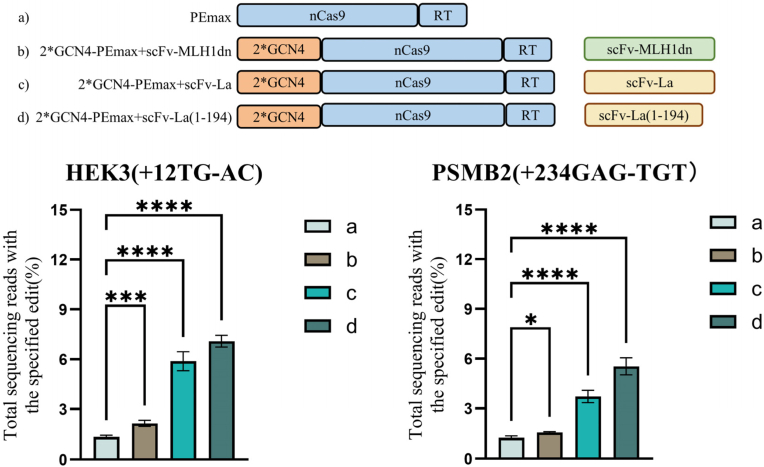
Prime editing efficiencies at multiple genomic loci using recruited MLH1dn, La(1–194), and La. The upper figure is the schematic illustration of the expression configurations with corresponding number, a to d. Bar graphs represent the mean values, and error bars indicate the standard deviation (SD) from three independent biological replicates. *P* values were calculated using one way analysis of variance (ANOVA), with ns indicating *P* > 0.05; ∗ indicating *P* ≤ 0.05; ∗∗ indicating *P* ≤ 0.01; ∗∗∗ indicating *P* ≤ 0.001; and ∗∗∗∗ indicating *P* ≤ 0.0001.

To determine whether the recruitment of specific cellular factors could enhance prime editing efficiency, we first employed the 2∗GCN4-PEmax system in combination with scFv-MLH1dn, scFv-La(1–194), or scFv-La plasmids to recruit MLH1dn, La(1–194), and La, respectively. Experiments were performed in HEK293T cells at two target loci, and the resulting editing efficiencies are shown in Fig. 2. At both the *HEK3* and *PSMB2* loci, recruitment of any of the three factors by the newly constructed 2∗GCN4-PEmax enhanced editing efficiency compared with the original PEmax editor. Specifically, recruitment of MLH1dn increased editing efficiency to 1.60-fold and 1.25-fold at *HEK3* and *PSMB2*, respectively; recruitment of La increased efficiency to 4.39-fold and 2.99-fold; and recruitment of La(1–194) resulted in the highest enhancement, 5.29-fold and 4.45-fold, respectively. These results indicate that recruitment of these cellular factors can effectively enhance prime editing efficiency compared with constructs lacking recruitment elements. Notably, recruitment of La(1–194) achieved the highest efficiency at both loci, and therefore La(1–194) was used for subsequent validation experiments.

### 2.2 Comparison of various expression configurations of the cellular factors

We further evaluated this in two aspects. First, we tested various approaches for freely overexpressing MLH1dn, including using a 2A peptide mediated co-expression and expression under EF1α or CMV promoters, which confirmed that recruiting MLH1dn is more effective than conventional non-recruited in enhancing prime editing efficiency. Second, we employed a 5∗GCN4-PE7 construct for recruitment, allowing up to five MLH1dn molecules to be recruited to the GCN4 repeats. This approach tested whether increasing the number of recruited MLH1dn molecules could further enhance prime editing efficiency.

**Fig. 3:**
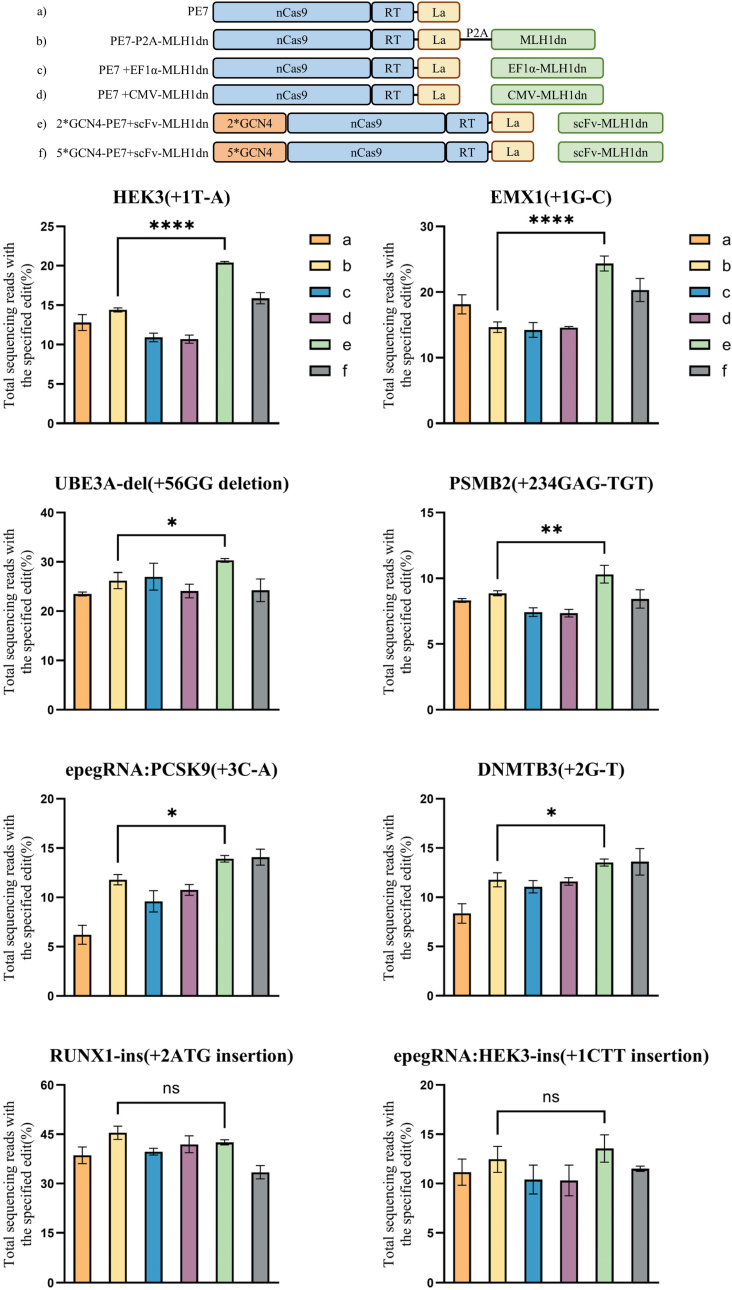
Prime editing efficiencies of various expression configurations of the cellular factors. The upper figure is the schematic illustration of the expression configurations with corresponding number, from a to f. Statistics are described in Fig. 2.

A comprehensive evaluation of prime editing efficiency for substitution, insertion, and deletion types was performed at eight genomic loci in HEK293T cells. As shown in Fig. 3, among the eight loci tested, five loci (*HEK3*, *EMX1*, *UBE3A*, *PSMB2*, and *PCSK9*) exhibited the highest editing efficiency when using 2∗GCN4-PE7 to recruit MLH1dn, showing a significant increase compared with PE7-P2A-MLH1dn, with an average improvement of 32%. The remaining three loci (*DNMTB3*, *RUNX1*, and epegRNA-*HEK3*) also displayed relatively high efficiencies. Specifically, at the *HEK3*, *EMX1*, *UBE3A*, *PSMB2*, and epegRNA-*PCSK9* loci, recruitment of MLH1dn by 2∗GCN4-PE7 increased prime editing efficiency by an average of 54.4% compared with PE7, by 32% compared with PE7-P2A-MLH1dn, by 51.2% compared with PE7 with EF1α-driven non-recruited of MLH1dn, and by 51% compared with PE7 with CMV-driven non-recruited of MLH1dn. Additionally, except for epegRNA-*PCSK9* and *DNMTB3*, 2∗GCN4-PE7-scFv-MLH1dn achieved higher editing efficiencies than 5∗GCN4-PE7-scFv-MLH1dn at the remaining six loci, with an average increase of 23.83%.

In summary, analysis across eight genomic loci demonstrates that the 2∗GCN4-PE7 system with recruited MLH1dn consistently achieves superior prime editing efficiency. MLH1dn recruitment is more effective than free overexpression in enhancing editing, and in most cases, the 2∗GCN4-PE7-scFv-MLH1dn system outperforms the 5∗GCN4-PE7-scFv-MLH1dn system. The reduced efficiency observed with 5∗GCN4-PE7 may reflect a threshold effect in factor recruitment, whereby excessive GCN4 repeats could introduce steric constraints or impair optimal assembly of the editing complex, as further discussed in the Discussion section.

### 2.3 Recruitment enhances editing efficiency in the PE7 system

**Fig. 4:**
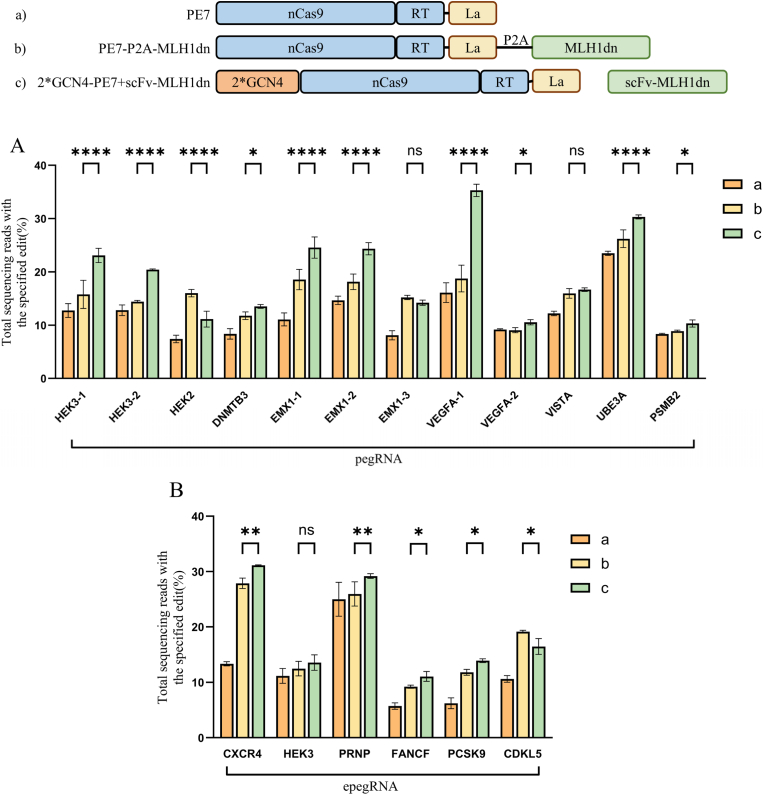
Enhanced prime editing across diverse genomic loci using pegRNAs (A) and epegRNAs (B) through MLH1dn recruitment. The upper figure is the schematic illustration of the expression configurations with corresponding number, from a to c. Statistics are described in Fig. 2.

Based on previous results, we selected the best-performing 2∗GCN4-PE7 system with recruited MLH1dn, designated PE7-scFv-MLH1dn, and compared it with the commonly used conventional system PE7-P2A-MLH1dn. As shown in Fig. 4, eighteen target sites were tested in HEK293T cells. Both PE7-scFv-MLH1dn and PE7-P2A-MLH1dn generally improved editing efficiencies relative to PE7 across the eighteen tested sites. While most loci exhibited clear enhancement, some sites, such as *VEGFA-2*, *PSMB2*, and epeg-*HEK3*, showed more modest gains. Detailed efficiencies for each site are described below. And PE7-scFv-MLH1dn overall outperformed PE7-P2A-MLH1dn.

Specifically, as shown in Fig. 4A, the editing efficiencies achieved by PE7-scFv-MLH1dn at the *HEK3*-1, *HEK3*-2, *HEK2*, *DNMTB3*, *EMX1*-1, *EMX1*-2, *EMX1*-3, *VEGFA*-1, *VEGFA*-2, *VISTA*, *UBE3A*, and *PSMB2* sites were 23.09, 35.3, 24.57, 10.54, 23.09, 20.4, 11.13, 13.52, 24.57, 24.34, 14.21, 35.3, 10.54, 16.65, 30.3, and 10.31, respectively, corresponding to 1.81-, 1.59-, 1.5-, 1.62-, 2.22-, 1.66-, 1.76-, 2.19-, 1.15-, 1.37-, 1.29-, and 1.24-fold improvements relative to PE7, with an average increase of 61.67%. Compared with the PE7-P2A-MLH1dn system, PE7-scFv-MLH1dn exhibited increased editing efficiencies of 1.46-, 1.42-, 0.7-, 1.15-, 1.32-, 1.34-, 0.94-, 1.88-, 1.17-, 1.04-, 1.16-, and 1.16-fold at the tested target sites; the recruitment-based strategy showed an average improvement of 22.84%.

In addition, we further validated this observation at six editing sites (*CXCR4*, *HEK3*, *PRNP*, *FANCF*, *PCSK9*, and *CDKL5*) using epegRNAs. As shown in Fig. 4B, both the PE7-P2A-MLH1dn system and the 2∗GCN4-PE7 system recruiting scFv-MLH1dn improved the editing efficiency of PE7. Among them, the editing efficiencies achieved by PE7-scFv-MLH1dn at the *CXCR4*, *HEK3*, *PRNP*, *FANCF*, *PCSK9*, and *CDKL5* sites were 31.12, 13.55, 29.18, 11.05, 13.9, and 16.46, respectively, corresponding to 2.33-, 1.22-, 1.17-, 1.94-, 2.24-, 1.55-, and 1.94-fold increases relative to PE7, with an average improvement of 74.12%. Compared with the PE7-P2A-MLH1dn system, PE7-scFv-MLH1dn exhibited increased editing efficiencies of 1.12-, 1.09-, 1.12-, 1.2-, 1.18-, and 0.86-fold at the tested target sites. Overall, the recruitment-based editing system showed higher editing efficiencies than the non-recruited PE7-P2A-MLH1dn system, with an average improvement of 9.5% over PE7-P2A-MLH1dn.

In summary, testing across various editing sites revealed that recruiting MLH1dn to PE7 via the SunTag system substantially enhanced prime editing efficiency, resulting in an average improvement of 67.9% relative to PE7 without any MLH1dn. Moreover, the PE7-scFv-MLH1dn achieved higher editing efficiencies than the PE7-P2A-MLH1dn system, with an average increase of 16.17% considering both pegRNA and epegRNA results. Notably, this enhancement was consistently observed across the tested targets, suggesting that the improved efficiency reflects a general effect rather than a preference for specific edit types. This global enhancement is consistent with the mechanism of MLH1dn, which inhibits the DNA mismatch repair pathway and thereby increases the retention of edited alleles across substitutions, insertions, and deletions.

### 2.4 Validation of PE7-scFv-MLH1dn editing efficiency in HeLa cells

Based on the above results obtained in HEK293T cells, we further evaluated the performance of the recruitment-based PE7-scFv-MLH1dn system in HeLa cells, which exhibit distinct transfection efficiency and DNA repair characteristics. Six genomic loci were selected for validation, and the editing efficiencies of PE7-scFv-MLH1dn were compared with those of PE7 and the conventional PE7-P2A-MLH1dn system.

**Fig. 5:**
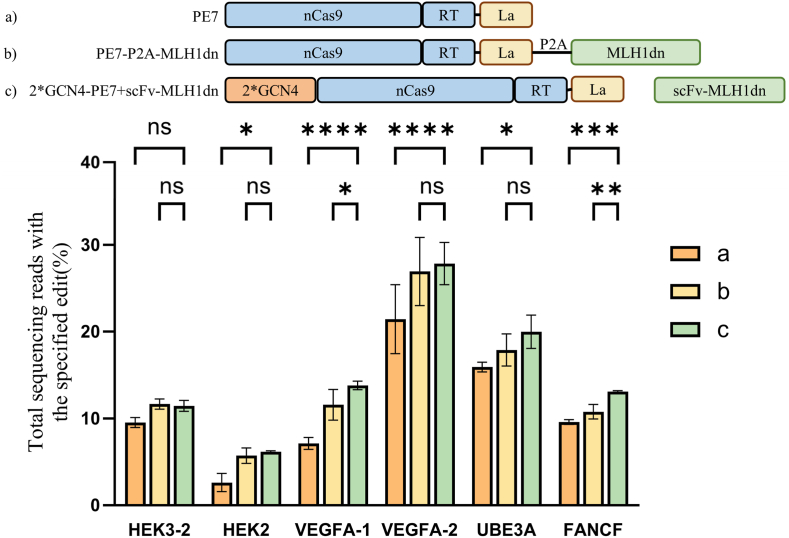
Validation of PE7-scFv-MLH1dn editing efficiency in HeLa cells. The upper figure is the schematic illustration of the expression configurations with corresponding number, from a to c. Statistics are described in Fig. 2.

As shown in Fig. 5, both PE7-scFv-MLH1dn and PE7-P2A-MLH1dn improved prime editing efficiencies relative to PE7 across all six tested loci in HeLa cells. Notably, PE7-scFv-MLH1dn consistently achieved higher editing efficiencies than PE7 at all target sites, with most loci exhibiting statistically significant improvements.

Specifically, the editing efficiencies achieved by PE7-scFv-MLH1dn at the *HEK3*-2, *HEK2*, *VEGFA*-1, *VEGFA*-2, *UBE3A*, and *FANCF* sites were 11.44, 6.17, 13.80, 27.85, 19.97, and 10.31, respectively, corresponding to 1.20-, 2.35-, 1.94-, 1.30-, 1.25-, and 1.36-fold increases relative to PE7, with an average improvement of 56.86%.When compared with the PE7-P2A-MLH1dn system, PE7-scFv-MLH1dn exhibited comparable or modestly higher editing efficiencies at most loci. Importantly, statistically significant improvements were observed at the *VEGFA*-1 and *FANCF* sites, whereas differences at the remaining loci did not reach statistical significance. Across all tested sites, PE7-scFv-MLH1dn showed fold changes of 0.98-, 1.07-, 1.19-, 1.03-, 1.11-, and 1.21-fold relative to PE7-P2A-MLH1dn, corresponding to an average improvement of 10.27%.

In summary, consistent with observations in HEK293T cells, recruitment of MLH1dn to PE7 via the SunTag system markedly enhanced prime editing efficiency relative to PE7 in HeLa cells. While the recruitment-based strategy achieved editing efficiencies largely comparable to those of the PE7-P2A-MLH1dn system, its significant advantages at specific loci (*VEGFA*-1 and *FANCF*), together with consistent improvements over PE7 across all tested sites, support the robustness of this approach in a cellular context distinct from HEK293T cells.

### 2.5 Assessment of the impact of MLH1dn recruitment on editing byproducts

**Fig. 6:**
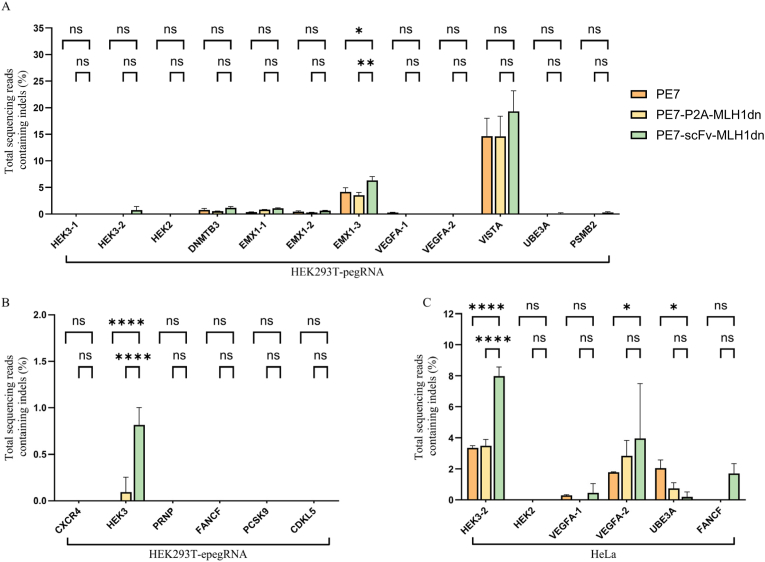
Assessment of unintended indel formation in HEK293T and HeLa cells. Indel frequencies were measured in both HEK293T and HeLa cells. (A) Indel frequencies at pegRNA-targeted loci in HEK293T cells. (B) Indel frequencies at epegRNA-targeted loci in HEK293T cells. (C) Indel frequencies at corresponding target loci in HeLa cells.

To examine whether recruitment of MLH1dn influences editing fidelity, we systematically quantified indel formation at multiple independent genomic loci in both HEK293T and HeLa cells. In HEK293T cells, indel frequencies induced by PE7-scFv-MLH1dn were low across most tested loci and were comparable to those observed with PE7, with values remaining close to background levels (Fig. 6A and B). However, a subset of loci exhibited clearly elevated indel frequencies, and several of these increases reached statistical significance.

A similar pattern was observed in HeLa cells. While indel levels at the majority of loci remained low and comparable between PE7-scFv-MLH1dn and PE7, certain loci showed modest to significant increases upon MLH1dn recruitment (Fig. 6C). Across both cell types, no large deletions, scaffold integrations, or other aberrant editing byproducts were detected, indicating that MLH1dn recruitment generally maintains genomic integrity.

Collectively, these results indicate that SunTag-mediated recruitment of MLH1dn enhances prime editing efficiency while inducing site-dependent increases in indel formation at a limited number of loci. These findings support the safety of localized MMR inhibition relative to global overexpression strategies, while highlighting the importance of locus-specific assessment in experimental design.

## 3 Discussion

Prime editing has rapidly evolved into one of the most versatile genome-engineering platforms (Supplementary Fig. 1), yet its broad application has been limited by variable and often suboptimal editing efficiencies across genomic loci, cell types, and edit classes. Numerous studies have demonstrated that cellular DNA repair pathways, particularly mismatch repair (MMR), play decisive roles in determining prime editing outcomes. In this study, we provide a systematic evaluation of how the spatial organization, expression modality, and recruitment efficiency of auxiliary factors influence prime editing outcomes. By integrating the SunTag-GCN4 scaffold into the PE7 architecture and employing scFv-mediated recruitment of MLH1dn (Supplementary Fig. 2), we established an optimized configuration, termed PE7-scFv-MLH1dn, that consistently enhances editing efficiency relative to PE7 and to non-recruited MLH1dn expression strategies, such as PE7-P2A-MLH1dn. (Fig. 1).

Our results suggest that simple overexpression of MLH1dn does not fully exploit its potential to modulate MMR at the editing site. Instead, local MLH1dn enrichment at the nicked DNA intermediate, achieved via the GCN4-scFv recruitment module, results in markedly higher editing efficiencies. This finding supports a mechanistic model in which prime editing is highly sensitive not only to the presence of MMR inhibition but also to the local stoichiometry and spatial proximity of MMR-modulating factors to the editing intermediate. This is consistent with prior observations that (i) MLH1dn increases prime editing through dominant-negative inhibition of MutLα and competitive binding with MSH2, and (ii) the La(1–194) domain fused to PEmax primarily stabilizes the pegRNA 3′tail, enhancing the efficiency of reverse transcription priming. By combining La-mediated pegRNA stabilization and MLH1dn-mediated localized MMR inhibition, PE7-scFv-MLH1dn effectively addresses two distinct molecular bottlenecks within the prime editing pathway. Furthermore, our comparison between 2∗GCN4 and 5∗GCN4 systems revealed that increasing the number of recruitment motifs does not necessarily yield further gains. This suggests that excessive clustering may introduce steric hindrance or impose a burden on protein folding and chromatin accessibility. Thus, the 2∗GCN4 stoichiometry appears to strike a favorable balance between recruitment strength and structural compatibility.

Testing across 18 genomic loci demonstrated that PE7-scFv-MLH1dn robustly improves efficiencies for substitution, insertion, and deletion edits, indicating broad applicability across edit types. Importantly, the enhancement was observed for both pegRNAs and epegRNAs, suggesting that the recruitment strategy is compatible with multiple pegRNA designs and can be directly integrated into PE workflows without requiring pegRNA redesign.

To assess the generalizability of the recruitment-based prime editing system beyond HEK293T cells, we evaluated the performance of PE7-scFv-MLH1dn in HeLa cells. PE7-scFv-MLH1dn consistently achieved higher editing efficiencies than PE7 across all tested loci, supporting the robustness of MLH1dn recruitment in a distinct cellular context. Compared with PE7-P2A-MLH1dn, PE7-scFv-MLH1dn showed a general trend toward increased editing efficiency, with statistically significant improvements observed at the *VEGFA*-1 and *FANCF* loci, while differences at other sites did not reach significance.

The target loci examined in HeLa cells were originally selected based on their strong responsiveness to MLH1dn-mediated enhancement in HEK293T cells, which may limit the observable advantage in a cell-type-specific DNA repair environment. As a cancer-derived cell line, HeLa cells exhibit pronounced genomic instability and altered DNA damage response and mismatch repair activities, factors that can influence both prime editing outcomes and the effectiveness of MMR inhibition strategies [28,29]. Despite these constraints, the consistent improvement over PE7 alone indicates that MLH1dn recruitment remains effective in promoting prime editing in HeLa cells. Together, these results support the robustness of the PE7-scFv-MLH1dn strategy while highlighting the influence of cell-type–specific DNA repair states on the magnitude of editing enhancement.

Together, these data reaffirm that manipulating local repair outcomes is more impactful than global MMR suppression, and that prime editing benefits from precise recruitment rather than indiscriminate overexpression of repair-modulating factors.

Given concerns that localized MMR inhibition might increase unintended edits, we systematically quantified indel formation at multiple independent loci in both HEK293T and HeLa cells. Overall, indel frequencies induced by PE7-scFv-MLH1dn were low at most loci and largely comparable to those observed with PE7; however, a subset of loci exhibited clearly elevated indel frequencies, with several sites reaching statistical significance. Importantly, no large deletions, scaffold integrations, or other aberrant editing byproducts were detected. These findings indicate that SunTag-mediated recruitment of MLH1dn enhances prime editing efficiency while maintaining high genomic fidelity, with site-dependent indel elevations that are limited in scope, supporting the safety of localized MMR inhibition relative to global overexpression strategies.

Recent advances such as PEmax, PE5/PE5max, PE7, MLH1dn co-delivery, and the small-molecule MLH1-SB reported by Park et al. further demonstrate that optimizing repair environment represents a central theme in improving prime editing. Our system differs from previous approaches in several key aspects. It provides sustained and locus-proximal MMR inhibition, which appears more potent than systemic MLH1dn expression. It offers modularity, as the SunTag cassette can be fused to PE editors of different architectures, enabling applications beyond PE7. Collectively, PE7-scFv-MLH1dn represents a complementary enhancement strategy that can be integrated with future editor variants, pegRNA stabilizing elements, or repair-pathway modulators.

Because prime editing is well-suited for therapeutic correction of pathogenic variants without inducing double-strand breaks, enhancing editing efficiency is crucial for feasibility in hard-to-edit cell types such as primary cells, iPSCs, and in vivo tissues. By achieving a substantial increase in editing efficiency, ranging from 16% to 70% depending on the locus, PE7-scFv-MLH1dn may enable more effective applications, including modeling human disease variants and therapeutic development, particularly for low-efficiency edit classes such as transversions and multi-nucleotide replacements.

Although PE7-scFv-MLH1dn offers notable improvement, several limitations remain. The SunTag based recruitment architecture substantially increases the overall coding size of the editor, exceeding the packaging capacity of commonly used viral vectors and thereby posing challenges for clinical delivery. Recruitment of MLH1dn may also influence genomic sites beyond the intended locus if ectopic tethering occurs, although this risk is likely lower than that associated with global MLH1dn overexpression. To address delivery constraints imposed by the large editor size, several alternative strategies are feasible, including split dual-AAV systems that reconstitute the editor in target cells [30,31], as well as non-viral approaches such as lipid nanoparticles for transient mRNA and pegRNA co-delivery. More advanced modalities, including LNP-mediated delivery of ribonucleoprotein complexes and engineered virus-like particles, could further reduce off-target risk while avoiding permanent genetic cargo integration [32,33]. Beyond delivery optimization, future improvements may come from combinatorial recruitment of additional cellular factors together with MLH1dn, temporal control of factor recruitment using inducible SunTag configurations to minimize unintended MMR suppression, and integration with next-generation PE or Cas variants to expand functional capabilities and editing scope. Together, these directions provide practical routes to overcome current size and safety limitations and facilitate clinical translation of enhanced prime editors.

In summary, our study demonstrates that precise spatial recruitment of MLH1dn in combination with La-enhanced pegRNA stabilization substantially improves prime editing outcomes. Through mechanistic tuning of repair factor localization, PE7-scFv-MLH1dn establishes a generalizable strategy for optimizing prime editing performance. These findings not only deepen our understanding of the molecular determinants of prime editing but also provide a powerful and modular improvement that can accelerate the application of prime editing in both basic research and therapeutic genome engineering.

## 4 Methods

### 4.1 Strains and culture conditions

As a cloning host, *E. coli* DH5α strains were cultured at 37 °C in L-broth medium (LB) with 1% (w/v) tryptone, 0.5% (w/v) yeast extract, and 1% (w/v) NaCl or L-agar (LA), consisting of LB medium with 1.5% agar. Ampicillin (100 mg/L) was added to the media as appropriate to ensure the accuracy of the screening result.

### 4.2 Plasmid construction

The plasmids of the SunTag system used in this study were constructed using Gibson assembly. The prime editor constructs are based on an engineered Cas9 nickase carrying the R221K, N394K, and H840A substitutions, which generates a site-specific single-strand break rather than a double-strand DNA break and is required for prime editing. All pegRNAs and epegRNAs were constructed using Golden Gate assembly, and PCR primers for pegRNA and epegRNA were designed with the desired sequences, including the PBS and RT template, embedded in the primers. Vector maps of key plasmids are provided in the Supplementary Materials.

### 4.3 Cell culture and transfection

HEK293T cells were obtained from ATCC and routinely tested for mycoplasma contamination. Cells were cultured in Dulbecco's minimal essential medium, supplemented with 10% (vol/vol) fetal bovine serum (FBS) and 1 × penicillin streptomycin. Cells were incubated and cultured at 37 °C with 5% CO_2_. Before transfection, the cells were seeded in 24-well plates, incubated for 16–18 h, and then transfected with PEI after reaching ∼40% confluence. A total of 600 ng of PE plasmid, 300 ng of pegRNA or pegRNA-expressing plasmid, and 200 ng of cellular factor plasmid were transfected with 60 μL of DMEM containing 3.3 μL of PEI. 24 h after transfection, 2 μg/mL puromycin was added to the medium and incubated for 4 days. Then, the cells were collected for high-throughput sequencing.

### 4.4 High-throughput sequencing and data analysis

Total genomic DNA was extracted using Quick Extract DNA extraction solution supplemented with proteinase K following the manufacturer's instructions with slight modifications. The samples were incubated at 55 °C for 10 min and inactivated at 80 °C for 3 min. Targeted regions (180–250 bp) of interest were amplified by PCR with Es Taq Master Mix and used for high-throughput DNA sequencing, as previously described. Libraries with different barcodes were analyzed by Illumina high-throughput sequencing. The data were split according to their barcodes, and the examined target sites were selected. Base substitution ratios were calculated by dividing base-substitution reads by total reads. The deep sequencing results were analyzed using CRISPResso2 (https://crispresso2.pinellolab.org/submission) [34].

### 4.5 Statistics and reproducibility

Statistical analyses were performed using GraphPad Prism 10. Data are presented as the mean ± SD from three independent experiments. Bar heights indicate mean values, and error bars represent the SD. *P*-values were calculated using a two-tailed unpaired *t*-test. *P* < 0.05 was considered statistically significant (∗*P* ≤ 0.05; ∗∗*P* ≤ 0.01; ∗∗∗*P* ≤ 0.001; ∗∗∗∗*P* ≤ 0.0001).

## CRediT authorship contribution statement

**Xuesong Li:** Writing – original draft, Validation, Software, Methodology, Investigation, Formal analysis, Data curation, Conceptualization. **Chaojun Qing:** Writing – original draft, Software, Methodology, Investigation, Formal analysis, Data curation, Conceptualization. **Bo Li:** Writing – review & editing, Writing – original draft, Supervision, Methodology, Investigation, Formal analysis. **Dongdong Zhao:** Resources, Project administration, Methodology, Investigation, Formal analysis, Data curation. **Peihua Li:** Validation, Supervision, Project administration, Methodology, Formal analysis, Data curation. **Wenzhu Tang:** Writing – review & editing, Visualization, Validation, Supervision, Software, Resources, Methodology. **Changhao Bi:** Writing – review & editing, Visualization, Validation, Supervision, Resources, Project administration, Funding acquisition, Formal analysis, Data curation, Conceptualization. **Xueli Zhang:** Visualization, Validation, Supervision, Resources, Project administration, Methodology, Funding acquisition.

## Declaration of competing interest

The authors declare that they have no known competing financial interests or personal relationships that could have appeared to influence the work reported in this paper.

## Data Availability

There is no restriction on data associated with this study. The DNA sequences of the main primers, as well as the individual components of sgRNA, pegRNA, and epegRNA are provided in Supplementary information. The high-throughput sequencing data generated in this study have been deposited in the NCBI database under accession code PRJNA1354970.

## References

[1] Makarova K.S., Wolf Y.I., Iranzo J., Shmakov S.A., Alkhnbashi O.S., Brouns S.J.J. (2020). Evolutionary classification of CRISPR–cas systems: a burst of class 2 and derived variants. Nat Rev Microbiol.

[2] Liang Z., Chen K., Li T., Zhang Y., Wang Y., Zhao Q. (2017). Efficient DNA-free genome editing of bread wheat using CRISPR/Cas9 ribonucleoprotein complexes. Nat Commun.

[3] Adli M. (2018). The CRISPR tool kit for genome editing and beyond. Nat Commun.

[4] Jinek M., Chylinski K., Fonfara I., Hauer M., Doudna J.A., Charpentier E. (2012). A programmable dual-RNA–guided DNA endonuclease in adaptive bacterial immunity. Science.

[5] Cong L., Ran F.A., Cox D., Lin S., Barretto R., Habib N. (2013). Multiplex genome engineering using CRISPR/cas systems. Science.

[6] Mali P., Yang L., Esvelt K.M., Aach J., Guell M., DiCarlo J.E. (2013). RNA-guided human genome engineering via Cas9. Science.

[7] Cho S.W., Kim S., Kim J.M., Kim J.-S. (2013). Targeted genome engineering in human cells with the Cas9 RNA-guided endonuclease. Nat Biotechnol.

[8] Komor A.C., Kim Y.B., Packer M.S., Zuris J.A., Liu D.R. (2016). Programmable editing of a target base in genomic DNA without double-stranded DNA cleavage. Nature.

[9] Nishida K., Arazoe T., Yachie N., Banno S., Kakimoto M., Tabata M. (2016). Targeted nucleotide editing using hybrid prokaryotic and vertebrate adaptive immune systems. Science.

[10] Gaudelli N.M., Komor A.C., Rees H.A., Packer M.S., Badran A.H., Bryson D.I. (2017). Programmable base editing of A•T to G•C in genomic DNA without DNA cleavage. Nature.

[11] Zhao D., Li J., Li S., Xin X., Hu M., Price M.A. (2021). Glycosylase base editors enable C-to-A and C-to-G base changes. Nat Biotechnol.

[12] Anzalone A.V., Randolph P.B., Davis J.R., Sousa A.A., Koblan L.W., Levy J.M. (2019). Search-and-replace genome editing without double-strand breaks or donor DNA. Nature.

[13] Chen P.J., Liu D.R. (2023). Prime editing for precise and highly versatile genome manipulation. Nat Rev Genet.

[14] Zhao Z., Shang P., Mohanraju P., Geijsen N. (2023). Prime editing: advances and therapeutic applications. Trends Biotechnol.

[15] Doudna J.A., Charpentier E. (2014). The new frontier of genome engineering with CRISPR-Cas9. Science.

[16] Chen P.J., Hussmann J.A., Yan J., Knipping F., Ravisankar P., Chen P.-F. (2021). Enhanced prime editing systems by manipulating cellular determinants of editing outcomes. Cell.

[17] Rahman M., Zulfiqar S., Raza M.A., Ahmad N., Zhang B. (2022). Engineering abiotic stress tolerance in crop plants through CRISPR genome editing. Cells.

[18] Ferreira da Silva J., Oliveira G.P., Arasa-Verge E.A., Kagiou C., Moretton A., Timelthaler G. (2022). Prime editing efficiency and fidelity are enhanced in the absence of mismatch repair. Nat Commun.

[19] Pellagatti A., Dolatshad H., Valletta S., Boultwood J. (2015). Application of CRISPR/Cas9 genome editing to the study and treatment of disease. Arch Toxicol.

[20] Nelson J.W., Randolph P.B., Shen S.P., Everette K.A., Chen P.J., Anzalone A.V. (2022). Engineered pegRNAs improve prime editing efficiency. Nat Biotechnol.

[21] Zhou Y., Liu Y., Hussmann J.A., Chen P.J. (2022). Enhancing prime editing via inhibition of mismatch repair pathway. Mol Biomed.

[22] Filippakopoulos P., Qi J., Picaud S., Shen Y., Smith W.B., Fedorov O. (2010). Selective inhibition of BET bromodomains. Nature.

[23] Chen R., Cao Y., Liu Y., Zhao D., Li J., Cheng Z. (2023). Enhancement of a prime editing system via optimal recruitment of the pioneer transcription factor P65. Nat Commun.

[24] Tanenbaum M.E., Gilbert L.A., Qi L.S., Weissman J.S., Vale R.D. (2014). A protein-tagging system for signal amplification in gene expression and fluorescence imaging. Cell.

[25] Mu S., Chen H., Li Q., Gou S., Liu X., Wang J. (2024). Enhancing prime editor flexibility with coiled-coil heterodimers. Genome Biol.

[26] Liu X., Cui S., Qi Q., Lei H., Zhang Y., Shen W. (2022). G-quadruplex-guided RNA engineering to modulate CRISPR-based genomic regulation. Nucleic Acids Res.

[27] Yan J., Oyler-Castrillo P., Ravisankar P., Ward C.C., Levesque S., Jing Y. (2024). Improving prime editing with an endogenous small RNA-binding protein. Nature.

[28] Negrini S., Gorgoulis V.G., Halazonetis T.D. (2010). Genomic instability - an evolving hallmark of cancer. Nat Rev Mol Cell Biol.

[29] Huang R., Zhou P.-K. (2021). DNA damage repair: historical perspectives, mechanistic pathways and clinical translation for targeted cancer therapy. Sig Transduct Target Ther.

[30] Zheng C., Liang S.-Q., Liu B., Liu P., Kwan S.-Y., Wolfe S.A. (2022). A flexible split prime editor using truncated reverse transcriptase improves dual-AAV delivery in mouse liver. Mol Ther.

[31] Qin H., Zhang W., Zhang S., Feng Y., Yao K. (2023). Vision rescue via unconstrained in vivo prime editing in degenerating neural retinas. J Exp Med.

[32] Hołubowicz R., Du S.W., Felgner J., Smidak R., Choi E.H., Palczewska G. (2025). Safer and efficient base editing and prime editing via ribonucleoproteins delivered through optimized lipid-nanoparticle formulations. Nat Biomed Eng.

[33] An M., Raguram A., Du S.W., Banskota S., Davis J.R., Newby G.A. (2024). Engineered virus-like particles for transient delivery of prime editor ribonucleoprotein complexes in vivo. Nat Biotechnol.

[34] Clement K., Rees H., Canver M.C., Gehrke J.M., Farouni R., Hsu J.Y. (2019). CRISPResso2 provides accurate and rapid genome editing sequence analysis. Nat Biotechnol.

