# Supplementary material for: Efficient PE system PE7-scFv-MLH1dn by optimizing the configuration of La and MLH1dn
Source: Synth Syst Biotechnol. 2026 May 25;14:342–50. doi: 10.1016/j.synbio.2026.04.035 (PMC13226125; doi:10.1016/j.synbio.2026.04.035)
Supplement: Multimedia component 1 [file mmc1.docx]

**Supplementary information** for

Efficient PE system PE7-scFv-MLH1dn by optimizing the configuration of

La and MLH1dn

Li, *et. al*

1. **Supplementary Fig. 1. Schematic representation of prime editing mechanism.**
2. **Supplementary Fig. 2. Construction design of Suntag-based prime editing systems.**
3. **Supplementary Fig. 3. Vector map of Pcmv-PEmax expression construct.**
4. **Supplementary Fig. 4. Vector map of Pcmv-PE7 construct.**
5. **Supplementary Fig. 5. Vector map of PE7-P2A-MLH1dn construct.**
6. **Supplementary Fig. 6. Vector map of 2*GCN4-PE construct.**
7. **Supplementary Fig. 7. Vector map of 5*GCN4-PE construct.**
8. **Supplementary Fig. 8. Vector map of scFv-MLH1dn construct.**
9. **Supplementary Table 1. pegRNA sequence.**
10. **Supplementary Table 2. epegRNA sequence.**
11. **Supplementary Table 3. Deep sequencing primers.**

**Supplemental figures**


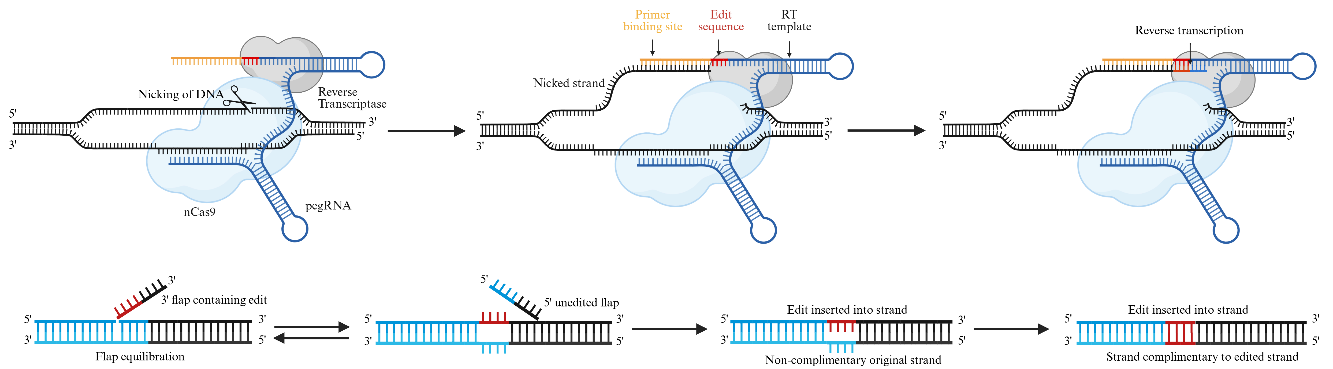


**Supplementary Fig. 1. Schematic representation of prime editing mechanism.**


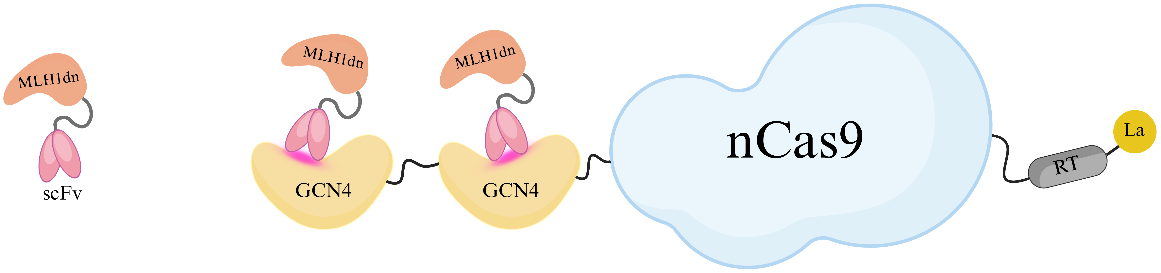


**Supplementary Fig. 2. Construction design of Suntag-based prime editing systems.**


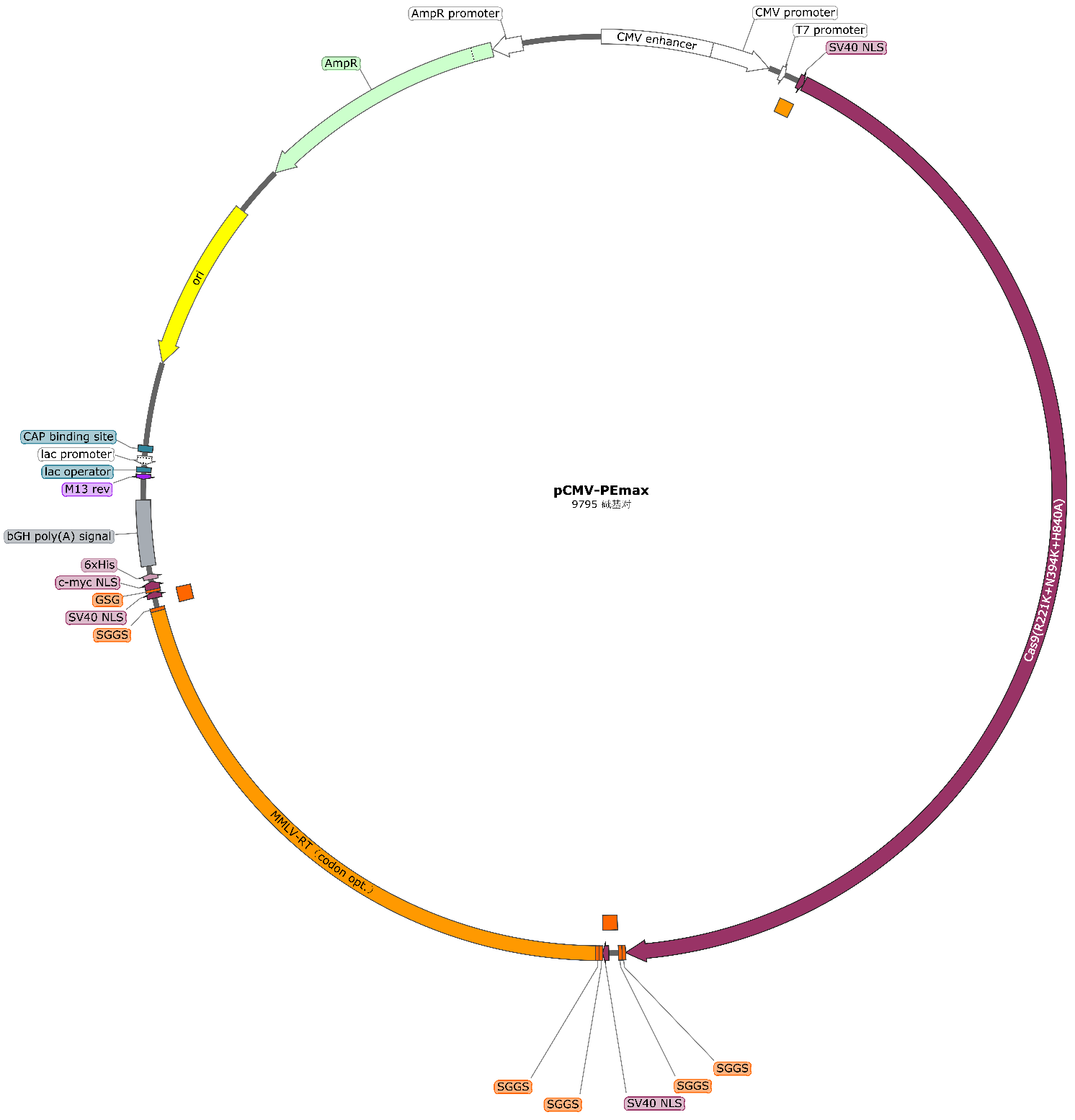


**Supplementary Fig. 3. Vector map of pCMV-PEmax expression construct.**


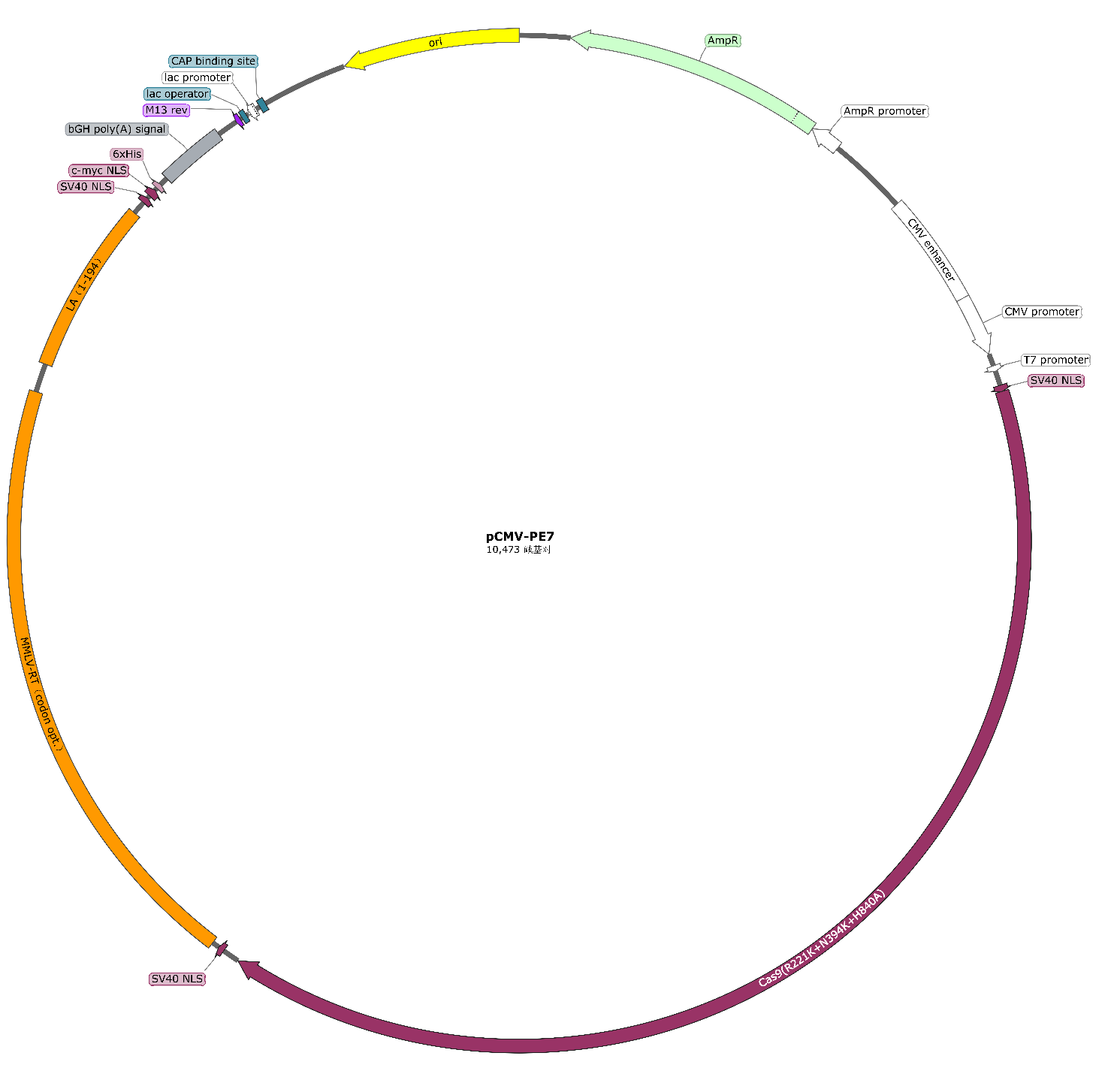


**Supplementary Fig. 4. Vector map of pCMV-PE7 construct.**


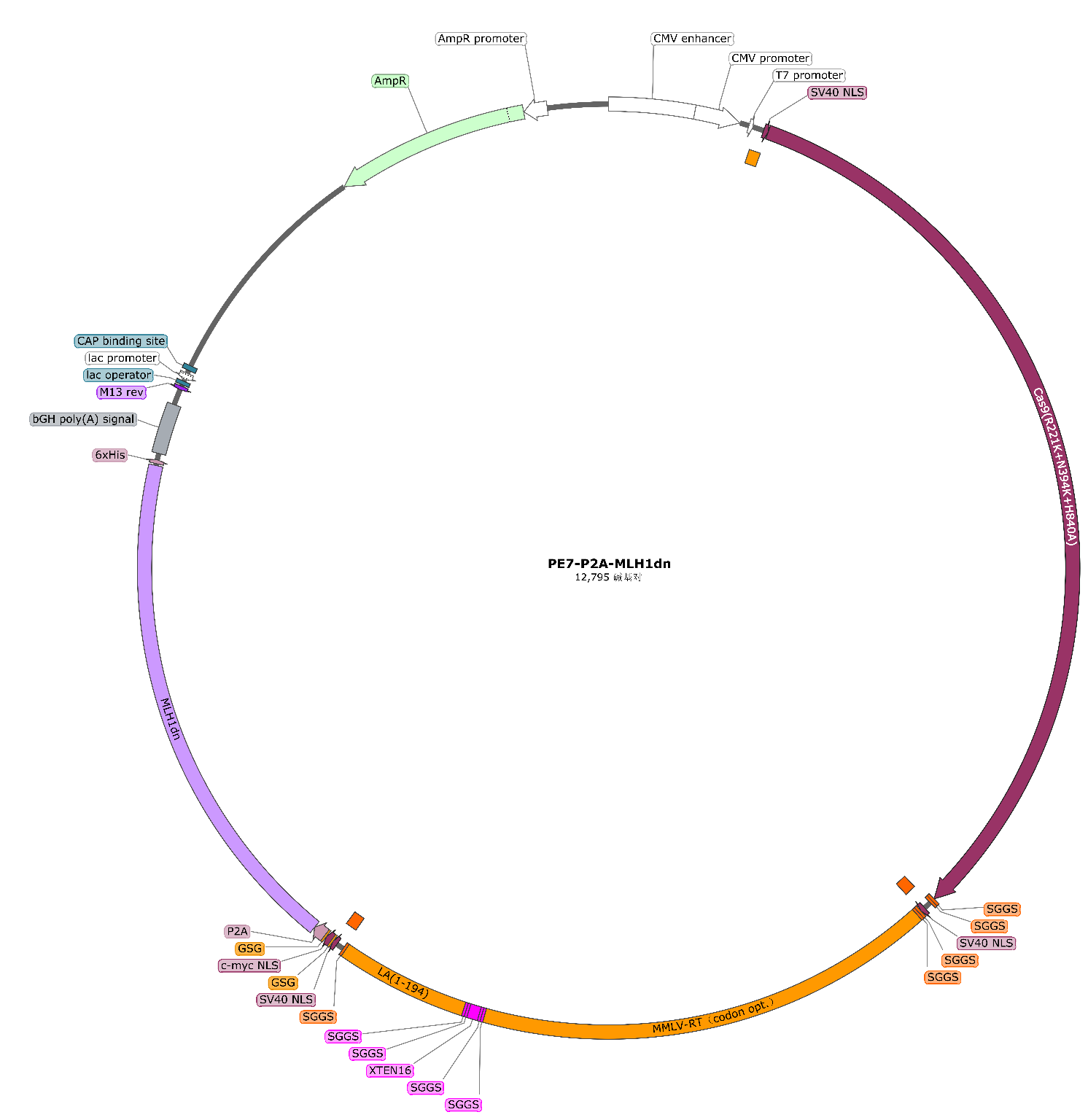


**Supplementary Fig. 5. Vector map of PE7-P2A-MLH1dn construct.**


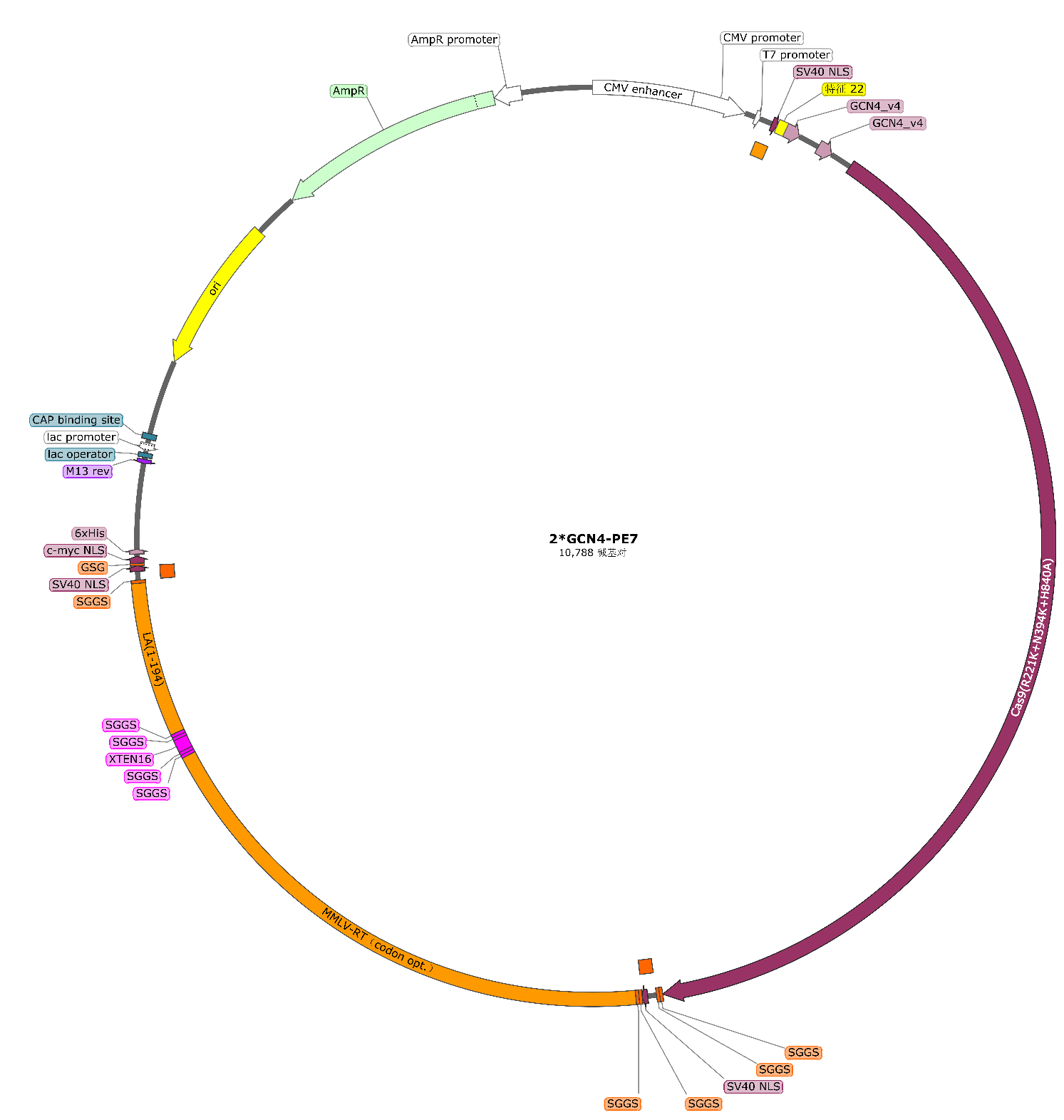


**Supplementary Fig. 6. Vector map of 2*GCN4-PE construct.**


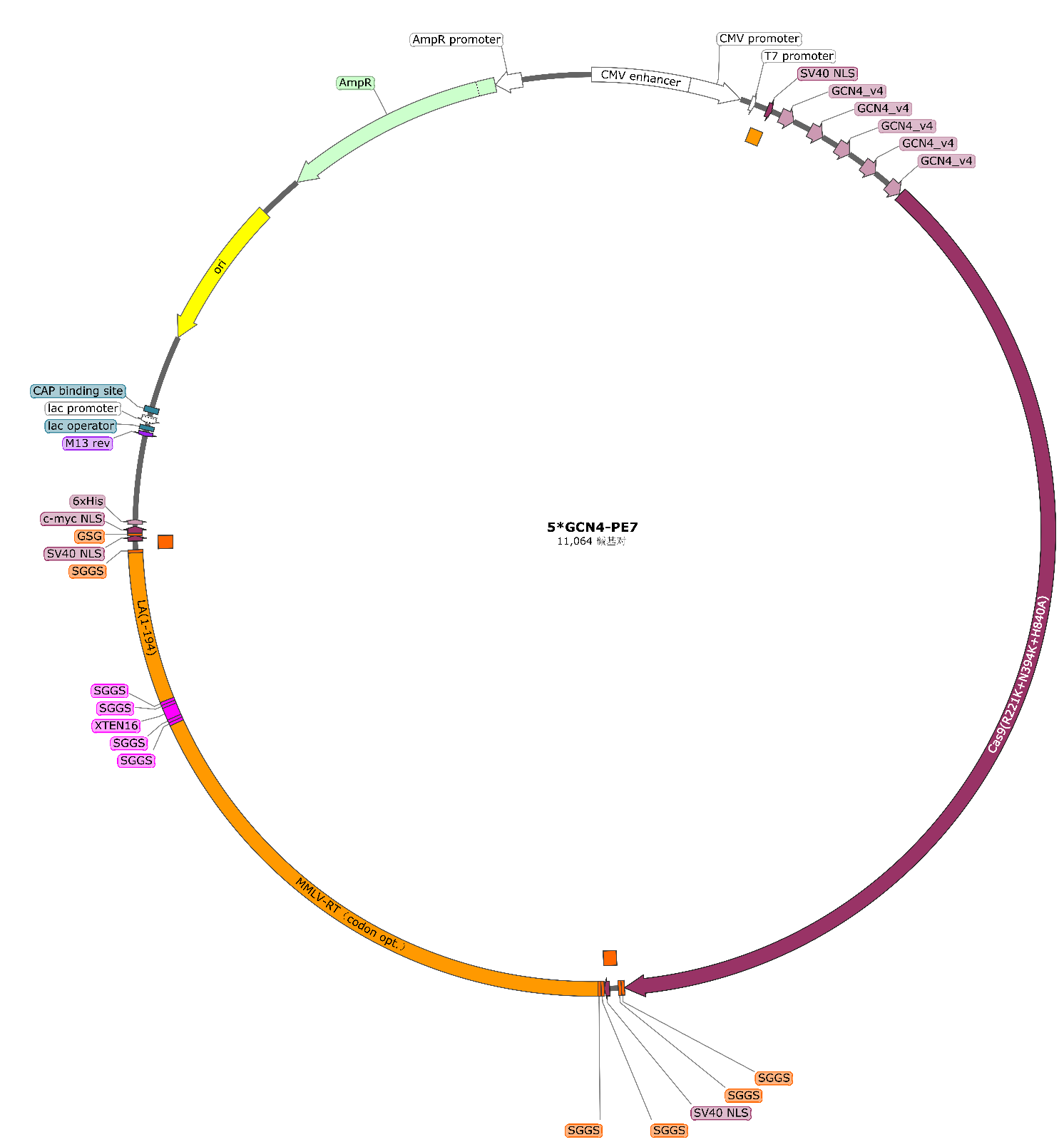


**Supplementary Fig 7. Vector map of 5*GCN4-PE construct.**


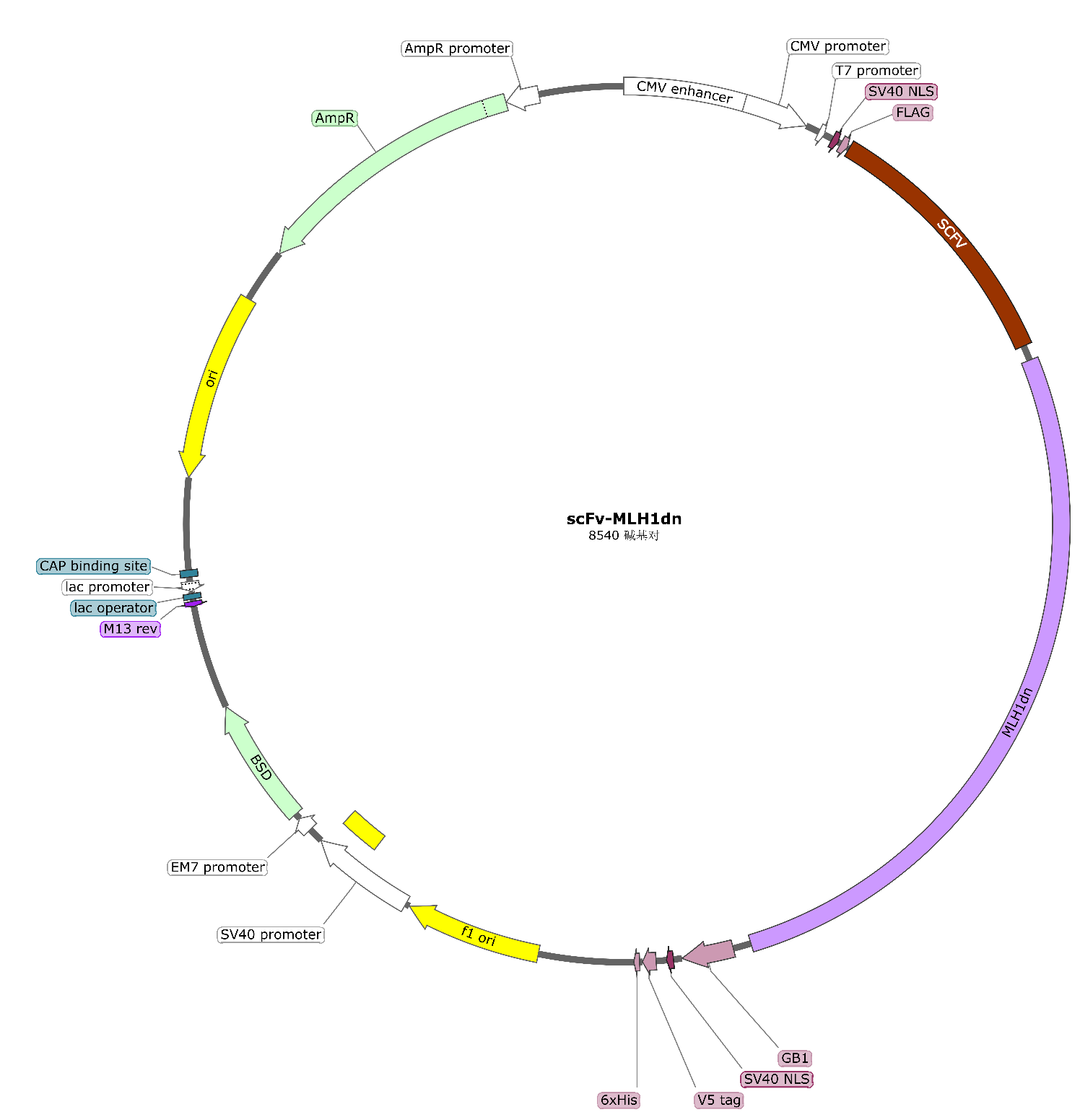


**Supplementary Fig. 8. Vector map of scFv-MLH1dn construct.**

**Supplementary Table 1. pegRNA sequence.**

| **pegRNA spacer** | **sequence** | **3’ extension sequence (5’-3’)** | **PBS**  **length(nt)** | **RT**  **Template**  **length (nt)** |
| --- | --- | --- | --- | --- |
| HEK3_site1_+12TG>AC | GGCCCAGACTGAGCACGTGA | TCTGCCATGTCGTGCTCAGTCTG | 13 | 10 |
| HEK3_site2_+1T>A | GGCCCAGACTGAGCACGTGA | TCCTCTGCCATCTCGTGCTCAGTCTG | 13 | 13 |
| HEK2_site3_+12CC>TT | GAATACTTCTTCAGAGTCCC | GAGTATCCTGAAACTCTGAAGAAGTAT | 15 | 12 |
| DNMTB3_+2G>T | GGCACTGCGGCTGGAGGTGG | TTAACCCCAACCTCCAGCCGC | 12 | 9 |
| EMX1_site1_+123CCC>TTT | GCTAACTTACTGTGTAACCC | TGACTTGCCCAAAATTACACAGTAAGTTA | 15 | 14 |
| EMX1_site2_+1G>C | GAGTCCGAGCAGAAGAAGAA | GTGATGGGAGCCCTTGTTCTTCTGCTCGG | 13 | 16 |
| EMX1_site3_+236CCG>TTT | GTATTCATTTCCCTAGCTCC | TAGCCGTCACTAAAGCTAGGGAAATGAATA | 16 | 14 |
| VEGFA_site1_+156CGG>TTT | GTCCCTCTTTAGCCAGAGCCG | TGCACAAACCGACTCTGGCTAAAGA | 13 | 12 |
| VEGFA_site2_+1T>A | GATGTCTGCAGGCCAGATGA | AATGTGCCATCTGGAGCCCTCTTCTGGCCTGCAGA | 13 | 22 |
| VEGFA_site3_+3A>C | GATGTCTGCAGGCCAGATGA | AATGTGCCATCTGGAGCCCGCATCTGGCCTGCAGA | 15 | 20 |
| UBE3A-3_del+56GG | GTACAGTTAGTACTCAGCAG | TTTGAGAGTACTGCTGAGTACTAACT | 16 | 10 |
| PSMB2_+234GAG>TGT | GTAAACAAAGCATAGACTGA | TTGTACCCACAAGTCTATGCTTTGTTT | 15 | 12 |
| RUNX1_+1ATGins | GCATTTTCAGGAGGAAGCGA | TGTCTGAAGCCATCCATGCTTCCTCCTGAAAAT | 16 | 17 |

**Supplementary Table 2. epegRNA sequence.**

| **epegRNA spacer** | **sequence** | **3’ extension sequence (5’- 3’)** | **PBS**  **length(nt)** | **RT**  **Template**  **length (nt)** |
| --- | --- | --- | --- | --- |
| CDKL5_+1C>T | GAGGGACTCCTAGAGGACTG | ATATTGACACAATTCCCCAATCCTCTAGGAGTC | 13 | 20 |
| CXCR4_+5G>C | GCAACCACCCACAAGTCATTG | TGACCGCTTCTACGCCAATGACTTGTGGGTGGT | 15 | 18 |
| FANCF_+3C>T | GGAATCCCTTCTGCAGCACC | GGAAAAGCGATCCAAGTGCTGCAGAAGGGAT | 14 | 17 |
| PCSK9_+3C>A | GCAGCCGTTGCCATCTGCTGC | GCCGGCTCCGTCAGCAGATGGCAACG | 13 | 13 |
| PRNP_+6G>T | GCAGTGGTGGGGGGCCTTGG | ATGTAGACGCCAAGGCCCCCCACC | 12 | 12 |
| HEK3_+1CTTins | GGCCCAGACTGAGCACGTGA | TCTGCCATCAAAGCGTGCTCAGTCTG | 13 | 13 |

**Supplementary Table 3. Deep sequencing primers.**

| **editing sites and Barcode** | **sequence (5'-3')** |  |
| --- | --- | --- |
| \| HEK3-site1-A1 \| \| --- \| \| HEK3-site1-A2 \| \| HEK3-site1-A3 \| \| HEK3-site1-A4 \| \| HEK3-site1-A5 \| \| HEK3-site1-A6 \| \| HEK3-site1-A7 \| \| HEK3-site1-A8 \| \| HEK3-site1-A9 \| \| HEK3-site1-reverse \| \| HEK3-site2-A1 \| \| HEK3-site2-A2 \| \| HEK3-site2-A3 \| \| HEK3-site2-A4 \| \| HEK3-site2-A5 \| \| HEK3-site2-A6 \| \| HEK3-site2-A7 \| \| HEK3-site2-A8 \| \| HEK3-site2-A9 \| \| HEK3-site2-reverse \| \| HEK3-site3-A1 \| \| HEK3-site3-A2 \| \| HEK3-site3-A3 \| \| HEK3-site3-A4 \| \| HEK3-site3-A5 \| \| HEK3-site3-A6 \| \| HEK3-site3-A7 \| \| HEK3-site3-A8 \| \| HEK3-site3-A9 \| \| HEK3-site3-reverse \| \| DNMTB3-A1 \| \| DNMTB3-A2 \| \| DNMTB3-A3 \| \| DNMTB3-A4 \| \| DNMTB3-A5 \| \| DNMTB3-A6 \| \| DNMTB3-A7 \| \| DNMTB3-A8 \| \| DNMTB3-A9 \| \| DNMTB3-reverse \| \| EMX1-site1--A1 \| \| EMX1-site1--A2 \| \| EMX1-site1--A3 \| \| EMX1-site1--A4 \| \| EMX1-site1--A5 \| \| EMX1-site1--A6 \| \| EMX1-site1--A7 \| \| EMX1-site1--A8 \| \| EMX1-site1--A9 \| \| EMX1-site1--reverse \| \| EMX1-site2-A1 \| \| EMX1-site2-A2 \| \| EMX1-site2-A3 \| \| EMX1-site2-A4 \| \| EMX1-site2-A5 \| \| EMX1-site2-A6 \| \| EMX1-site2-A7 \| \| EMX1-site2-A8 \| \| EMX1-site2-A9 \| \| EMX1-site2-reverse \| \| EMX1-site3-A1 \| \| EMX1-site3-A2 \| \| EMX1-site3-A3 \| \| EMX1-site3-A4 \| \| EMX1-site3-A5 \| \| EMX1-site3-A6 \| \| EMX1-site3-A7 \| \| EMX1-site3-A8 \| \| EMX1-site3-A9 \| \| EMX1-site3-reverse \| \| VEGFA-site1-A1 \| \| VEGFA-site1-A2 \| \| VEGFA-site1-A3 \| \| VEGFA-site1-A4 \| \| VEGFA-site1-A5 \| \| VEGFA-site1-A6 \| \| VEGFA-site1-A7 \| \| VEGFA-site1-A8 \| \| VEGFA-site1-A9 \| \| VEGFA-site1-reverse \| \| VEGFA-site2-A1 \| \| VEGFA-site2-A2 \| \| VEGFA-site2-A3 \| \| VEGFA-site2-A4 \| \| VEGFA-site2-A5 \| \| VEGFA-site2-A6 \| \| VEGFA-site2-A7 \| \| VEGFA-site2-A8 \| \| VEGFA-site2-A9 \| \| VEGFA-site2-reverse \| \| VEGFA-site3-A1 \| \| VEGFA-site3-A2 \| \| VEGFA-site3-A3 \| \| VEGFA-site3-A4 \| \| VEGFA-site3-A5 \| \| VEGFA-site3-A6 \| \| VEGFA-site3-A7 \| \| VEGFA-site3-A8 \| \| VEGFA-site3-A9 \| \| VEGFA-site3-reverse \| \| UBE3A-3-A1 \| \| UBE3A-3-A2 \| \| UBE3A-3-A3 \| \| UBE3A-3-A4 \| \| UBE3A-3-A5 \| \| UBE3A-3-A6 \| \| UBE3A-3-A7 \| \| UBE3A-3-A8 \| \| UBE3A-3-A9 \| \| UBE3A-3-reverse \| \| PSMB2-A1 \| \| PSMB2-A2 \| \| PSMB2-A3 \| \| PSMB2-A4 \| \| PSMB2-A5 \| \| PSMB2-A6 \| \| PSMB2-A7 \| \| PSMB2-A8 \| \| PSMB2-A9 \| \| PSMB2-reverse \| \| RUNX1-A1 \| \| RUNX1-A2 \| \| RUNX1-A3 \| \| RUNX1-A4 \| \| RUNX1-A5 \| \| RUNX1-A6 \| \| RUNX1-A7 \| \| RUNX1-A8 \| \| RUNX1-A9 \| \| RUNX1-reverse \| \| CDKL5-A1 \| \| CDKL5-A2 \| \| CDKL5-A3 \| \| CDKL5-A4 \| \| CDKL5-A5 \| \| CDKL5-A6 \| \| CDKL5-A7 \| \| CDKL5-A8 \| \| CDKL5-A9 \| \| CDKL5-reverse \| \| CXCR4-A1 \| \| CXCR4-A2 \| \| CXCR4-A3 \| \| CXCR4-A4 \| \| CXCR4-A5 \| \| CXCR4-A6 \| \| CXCR4-A7 \| \| CXCR4-A8 \| \| CXCR4-A9 \| \| CXCR4-reverse \| \| FANCF-A1 \| \| FANCF-A2 \| \| FANCF-A3 \| \| FANCF-A4 \| \| FANCF-A5 \| \| FANCF-A6 \| \| FANCF-A7 \| \| FANCF-A8 \| \| FANCF-A9 \| \| FANCF-reverse \| \| PCSK9-A1 \| \| PCSK9-A2 \| \| PCSK9-A3 \| \| PCSK9-A4 \| \| PCSK9-A5 \| \| PCSK9-A6 \| \| PCSK9-A7 \| \| PCSK9-A8 \| \| PCSK9-A9 \| \| PCSK9-reverse \| \| PRNP-A1 \| \| PRNP-A2 \| \| PRNP-A3 \| \| PRNP-A4 \| \| PRNP-A5 \| \| PRNP-A6 \| \| PRNP-A7 \| \| PRNP-A8 \| \| PRNP-A9 \| \| PRNP-reverse \| \| HEK3-A1 \| \| HEK3-A2 \| \| HEK3-A3 \| \| HEK3-A4 \| \| HEK3-A5 \| \| HEK3-A6 \| \| HEK3-A7 \| \| HEK3-A8 \| \| HEK3-A9 \| \| HEK3-reverse \| | \| TGTGCTAGTCTtaagcatgcatttgtaggcttg \| \| --- \| \| CGTGCTATCGCtaagcatgcatttgtaggcttg \| \| AGAGCTGATCGtaagcatgcatttgtaggcttg \| \| CCATCAGTGCTtaagcatgcatttgtaggcttg \| \| CGATGAGTGCTtaagcatgcatttgtaggcttg \| \| ACACATGTGCTtaagcatgcatttgtaggcttg \| \| TCGTATGTGCTtaagcatgcatttgtaggcttg \| \| GGAAGTGTGCTtaagcatgcatttgtaggcttg \| \| GCATCAGTGCTtaagcatgcatttgtaggcttg \| \| ttccagcccagccaaacttg \| \| TAAACTATTACtaagcatgcatttgtaggcttg \| \| AGACGAGTGGTtaagcatgcatttgtaggcttg \| \| TGGGTCTTCTTtaagcatgcatttgtaggcttg \| \| GTATCTTGTTTtaagcatgcatttgtaggcttg \| \| TCAGGAAGGCCtaagcatgcatttgtaggcttg \| \| CCTAGTGAGAGtaagcatgcatttgtaggcttg \| \| CATTCTCCCGGtaagcatgcatttgtaggcttg \| \| TCTTGTACACTtaagcatgcatttgtaggcttg \| \| GAGCATTCACAtaagcatgcatttgtaggcttg \| \| ttccagcccagccaaacttg \| \| TACCACATGCCtctgggagtgcagtaatg \| \| GTAATAGCGGCtctgggagtgcagtaatg \| \| TCTCAAGCACCtctgggagtgcagtaatg \| \| CCGTAGCGACGtctgggagtgcagtaatg \| \| GCACGTAGAGTtctgggagtgcagtaatg \| \| GTATGCACTATtctgggagtgcagtaatg \| \| GGCCTCTATCGtctgggagtgcagtaatg \| \| GATCTGACTGCtctgggagtgcagtaatg \| \| ATGCAACCGCGtctgggagtgcagtaatg \| \| cctcccttagagttaagc \| \| AGAACAGATGGggcgaggcagagggtccaaa \| \| GGCTCAGGCGGggcgaggcagagggtccaaa \| \| TTGAGGCGGCGggcgaggcagagggtccaaa \| \| CCGTGCGTACGggcgaggcagagggtccaaa \| \| CAGAAGCGAAGggcgaggcagagggtccaaa \| \| GCCATGACCGTggcgaggcagagggtccaaa \| \| GTGACAATGTGggcgaggcagagggtccaaa \| \| TCCAACATTCGggcgaggcagagggtccaaa \| \| GAGACATCTGCggcgaggcagagggtccaaa \| \| tttcccgggctcctttcaacc \| \| CACTACGCTCCgacagagcttcccaggtgggcaaac \| \| CGTACTGACGAgacagagcttcccaggtgggcaaac \| \| TGTGCTGTCTCgacagagcttcccaggtgggcaaac \| \| CTCTAGACAGCgacagagcttcccaggtgggcaaac \| \| GCAGAGCCAGTgacagagcttcccaggtgggcaaac \| \| CAATACGCGCAgacagagcttcccaggtgggcaaac \| \| TCGCATGTAAGgacagagcttcccaggtgggcaaac \| \| CTAAGGCGTCTgacagagcttcccaggtgggcaaac \| \| GGTCTAAGACTgacagagcttcccaggtgggcaaac \| \| gctactaatctgacatacta \| \| TCAGTCGTGCTgacaaagtacaaacggcaga \| \| CACGTAGTGCTgacaaagtacaaacggcaga \| \| CAGAGCATGCTgacaaagtacaaacggcaga \| \| CACAGTGTGCTgacaaagtacaaacggcaga \| \| CATGCTGTGCTgacaaagtacaaacggcaga \| \| CTGCTCGTGCTgacaaagtacaaacggcaga \| \| CTCAGAGTGCTgacaaagtacaaacggcaga \| \| CTCTGCATGCTgacaaagtacaaacggcaga \| \| CTAGCTGTGCTgacaaagtacaaacggcaga \| \| agcagcactctgccctcgt \| \| TGATGGGAGAGccctcattcctgggaatctcttg \| \| ACCAGATTGACccctcattcctgggaatctcttg \| \| ACGACTGAATCccctcattcctgggaatctcttg \| \| AGCTTGTTGCTccctcattcctgggaatctcttg \| \| AGTACAGGTTAccctcattcctgggaatctcttg \| \| ACTCCGTCGGGccctcattcctgggaatctcttg \| \| AGAACGGCTCTccctcattcctgggaatctcttg \| \| AGTTAGTTATCccctcattcctgggaatctcttg \| \| TGTGCAAGACTccctcattcctgggaatctcttg \| \| cagtttcggactaggacagttagc \| \| TCCTCGCTGAAcacattgtcagagggacacactg \| \| TCGGCATCATGcacattgtcagagggacacactg \| \| TGCGAGAGCACcacattgtcagagggacacactg \| \| TCAGCCGCCTTcacattgtcagagggacacactg \| \| TCGATGCTAGGcacattgtcagagggacacactg \| \| CAGGAGCGTTTcacattgtcagagggacacactg \| \| CGACAGGTGCAcacattgtcagagggacacactg \| \| CACCGTTATGAcacattgtcagagggacacactg \| \| CCATCAATGCCcacattgtcagagggacacactg \| \| cccagcttccctgtggtg \| \| TCAGGACGCTCttcccaaaggaccccagtca \| \| CGATCAGAGATTtcccaaaggaccccagtca \| \| GAGATAATTCATtcccaaaggaccccagtca \| \| ATGCAGGAATCTtcccaaaggaccccagtca \| \| GAATGCCTGACTtcccaaaggaccccagtca \| \| CTGATGACATGTtcccaaaggaccccagtca \| \| GCATGCGAGCTTtcccaaaggaccccagtca \| \| GCGCTATCCGCTtcccaaaggaccccagtca \| \| CGTCTATCCGCTtcccaaaggaccccagtca \| \| gacctcccaaacagctacat \| \| CACTATTACTTttcccaaaggaccccagtca \| \| GACACGCGTGTttcccaaaggaccccagtca \| \| TCGAGATATCTttcccaaaggaccccagtca \| \| CCTGCGTGCAGttcccaaaggaccccagtca \| \| GAGGTACAGAGttcccaaaggaccccagtca \| \| CAGAATGTAGCttcccaaaggaccccagtca \| \| TAAGAATTAATttcccaaaggaccccagtca \| \| TCCTTGTTCTGttcccaaaggaccccagtca \| \| ACCGAGGTGCTttcccaaaggaccccagtca \| \| gacctcccaaacagctacat \| \| GTCGGATTAACcagcttactgcccctctttc \| \| GACGAGTCTGTcagcttactgcccctctttc \| \| CATAGGCACGGcagcttactgcccctctttc \| \| AACAGACTGATcagcttactgcccctctttc \| \| GATCCAACCACcagcttactgcccctctttc \| \| AACACGGCGAAcagcttactgcccctctttc \| \| TTGGCAATCGAcagcttactgcccctctttc \| \| CATACGCACACcagcttactgcccctctttc \| \| TCAATGTGCCAcagcttactgcccctctttc \| \| tcagaatcaagagatcagagctgag \| \| CAGAGGCCGATagctgttgcatgaggaaagg \| \| CACTTTGCTCCagctgttgcatgaggaaagg \| \| CATGCTTTCGAagctgttgcatgaggaaagg \| \| CTCAGACGGTGagctgttgcatgaggaaagg \| \| CTTTGCGACCAagctgttgcatgaggaaagg \| \| CTAGCTGGTGTagctgttgcatgaggaaagg \| \| AGGATCGGAGTagctgttgcatgaggaaagg \| \| CAGAGCAAAGAagctgttgcatgaggaaagg \| \| AGTGCTTCTGGagctgttgcatgaggaaagg \| \| gccgtgggagacaattcatatc \| \| TGAGTTGGCAGcgctccgaaggtaaaagaaatc \| \| TGTCAGAGATGcgctccgaaggtaaaagaaatc \| \| CTTGTTCTTGGcgctccgaaggtaaaagaaatc \| \| CCATGTACCGCcgctccgaaggtaaaagaaatc \| \| TCGGTGCGTCAcgctccgaaggtaaaagaaatc \| \| TGAAGACAATAcgctccgaaggtaaaagaaatc \| \| TCGACACGTACcgctccgaaggtaaaagaaatc \| \| GCGCGTTGCAAcgctccgaaggtaaaagaaatc \| \| GATGAAGCTATcgctccgaaggtaaaagaaatc \| \| acaagctgccatttcattacag \| \| TCCGGTGCTCAggttgctcacagacttggag \| \| GATGGCAGATCggttgctcacagacttggag \| \| AGTCCGCTCTCggttgctcacagacttggag \| \| ATACGTGTAGGggttgctcacagacttggag \| \| CCTTGTTCCTCggttgctcacagacttggag \| \| ATACGCCAGTGggttgctcacagacttggag \| \| AGAGTGATCCTggttgctcacagacttggag \| \| ATCGCCGCGCTggttgctcacagacttggag \| \| GGTTAACGCAGggttgctcacagacttggag \| \| ctcaagtcaaacagcagatc \| \| GAGGACTATCAggatgacaataccaggcagg \| \| TGTAATACGGTggatgacaataccaggcagg \| \| CACCATATTCGggatgacaataccaggcagg \| \| CTACTATTGGCggatgacaataccaggcagg \| \| TGTCACAGAGGggatgacaataccaggcagg \| \| CTCGCGTCCAGggatgacaataccaggcagg \| \| CACCATTAAGCggatgacaataccaggcagg \| \| GTGTGTCACCTggatgacaataccaggcagg \| \| GAGTTGGACTCggatgacaataccaggcagg \| \| gaagctgttggctgaaaagg \| \| GGTGGCGAACAcgccgtctccaaggtgaaa \| \| CCACGCGCTCGcgccgtctccaaggtgaaa \| \| GCGCTGTGTAAcgccgtctccaaggtgaaa \| \| CCTGCCATATTcgccgtctccaaggtgaaa \| \| TTTGTAGTTTAcgccgtctccaaggtgaaa \| \| ATAACCATTGAcgccgtctccaaggtgaaa \| \| GCGCCAGCGAAcgccgtctccaaggtgaaa \| \| ATCGATAGCGCcgccgtctccaaggtgaaa \| \| AAGCTAGCTGAcgccgtctccaaggtgaaa \| \| cgatggatgtggcgcaggta \| \| GCCGACGTTCCcgtagacaacacgtgtgtag \| \| TGAAGCTTCCGcgtagacaacacgtgtgtag \| \| ATACCTCAACCcgtagacaacacgtgtgtag \| \| TCAATACGCTGcgtagacaacacgtgtgtag \| \| AGATATTACGCcgtagacaacacgtgtgtag \| \| GCTGCACCGTCcgtagacaacacgtgtgtag \| \| TCCATTCTGGTcgtagacaacacgtgtgtag \| \| CAGAGCGGATCcgtagacaacacgtgtgtag \| \| CTCGGAAGGTGcgtagacaacacgtgtgtag \| \| gagagggacaagtcggaacc \| \| ATTCTGATAACgtggcacccacagtcagtgg \| \| AAGGCGATAATgtggcacccacagtcagtgg \| \| TCCGCTTGTGCgtggcacccacagtcagtgg \| \| CAGCGCTATATgtggcacccacagtcagtgg \| \| AGCTCTGATGTgtggcacccacagtcagtgg \| \| CGCCGTGTCGAgtggcacccacagtcagtgg \| \| GCATGATTGGTgtggcacccacagtcagtgg \| \| GTTGTCAGATCgtggcacccacagtcagtgg \| \| TAACAGTATTGgtggcacccacagtcagtgg \| \| tccatgggcctgtagtacac \| \| GGTTAGTACAAtaagcatgcatttgtaggcttg \| \| TTAAACTAATTtaagcatgcatttgtaggcttg \| \| GAGTTATATACtaagcatgcatttgtaggcttg \| \| AGGGTACAACCtaagcatgcatttgtaggcttg \| \| ATTCTCCGAGTtaagcatgcatttgtaggcttg \| \| CTGGGTACTAGtaagcatgcatttgtaggcttg \| \| TTATCTGAAAGtaagcatgcatttgtaggcttg \| \| TGATCAACCCGtaagcatgcatttgtaggcttg \| \| CACTCCGTACAtaagcatgcatttgtaggcttg \| \| ttccagcccagccaaacttg \| | |
